# Role of NEDD9 in invasion and metastasis of lung adenocarcinoma

**DOI:** 10.3892/etm.2012.693

**Published:** 2012-09-03

**Authors:** JING-XIA CHANG, FENG GAO, GUO-QIANG ZHAO, GUO-JUN ZHANG

**Affiliations:** 1 Department of Respiratory Medicine, First Affiliated Hospital of Zhengzhou University;; 2 Department of Microorganisms and Immunization, Preclinical Medicine, Zhengzhou University, Zhengzhou, Henan, P.R. China

**Keywords:** NEDD9, lung adenocarcinoma, metastases, immunohistochemistry, fluorescence quantitative reverse transcription-polymerase chain reaction

## Abstract

Treatment failure for lung adenocarcinoma is frequently due to lymph node metastasis and invasion to neighboring organs. The aim of the present study was to investigate the invasion- and metastasis-related gene, neural precursor cell expressed, developmentally downregulated 9 (NEDD9), in lung adenocarcinoma tissues and cell lines. The expression of NEDD9 was analyzed by the SP method of immunohistochemistry for 60 formalin-fixed and paraffin-embedded (FFPE) lung adenocarcinoma tissues in which 32 cases were metastastic and 28 were without metastases. NEDD9 mRNA expression and protein levels were quantified by fluorescence quantitative reverse transcription-polymerase chain reaction (FQ-PCR) and western blotting in the highly invasive lung adenocarcinoma cell lines A549 and 95D as well as in SPC-A-1 cells with low invasive potential. The immunostaining scores revealed a statistically significant difference between metastatic and non-metastatic lung adenocarcinomas (p<0.001). FQ-PCR and western blotting demonstrated that NEDD9 expression was higher in A549 and 95D compared to SPC-A-1 cells (P=0.003). Our results provide evidence that NEDD9 is upregulated in metastatic lung adenocarcinoma and in highly invasive lung adenocarcinoma cell lines, suggesting its potential involvement in regulating cell migration and invasion.

## Introduction

NEDD9, also known as HEF1 and Cas-L (1,2), was initially identified by its developmentally regulated expression pattern in early embryonic, but not adult, mouse brain (3). NEDD9 resides at focal adhesions and, as such, is positioned to interact with a number of the key proteins coordinating migration (4). In the past six years, studies have identified elevated NEDD9 expression as contributing to cancer metastasis in multiple cancer types; it is an attractive biomarker of metastatic melanomas (5). Whether this is true for other types of cancers remains to be established; for example, reduced levels of NEDD9 transcripts characterize an MDA-MB-231 breast cancer cell line selected by serial *in vivo* passages for efficient metastasis to the lung in mice (6).

Lung cancer, in particular lung adenocarcinoma, is the leading cause of cancer-related mortality worldwide (7). Most mortality associated with cancer arises from uncontrolled metastases; thus, a better understanding of the properties of proteins specifically associated with promoting this process may yield insights that improve cancer diagnosis and treatment. NEDD9 is a necessary and specific downstream effector of focal adhesion kinase (FAK) that promotes the migration of glioblastoma cells (8). Carelli and colleagues found that FAK is upregulated in non-small cell lung cancer (NSCLC), thereby suggesting its potential involvement in lung cancer progression (9).

Overexpression of the NEDD9 protein has been strongly linked to poor prognosis and increased metastasis in cancer, as well as resistance to first-line chemotherapeutics in multiple tumor types. Its upregulation may play a role in the tumorigenesis of invasive tumors, but its involvement in human lung adenocarcinoma tissues has yet to be determined. In our study, we immunohistochemically compared NEDD9 expression and localization in 60 FFPE lung adenocarcinoma tissues and analyzed NEDD9 mRNA and protein levels in three invasive lung adenocarcinoma cell lines, and also investigated the expression and clinical significance of NEDD9 in 60 surgically resected stage I through IV lung adenocarcinomas with known clinicopathological features.

## Materials and methods

### Tissue collection

For immunostaining of NEDD9, archival paraffin blocks of pulmonary specimens from 60 lung adenocarcinoma patients were collected from the Department of Pathology in our hospital. All lung adenocarcinoma cases were clinically and pathologically proven, without having previously received chemotherapy or radiation therapy. The protocols used in the studies were approved by the Hospital’s Protection of Human Subjects Committee. Informed consent was obtained from all patients enrolled in the study.

Tumor histotype was determined according to the WHO classification of lung and pleural tumours (1999) and TNM staging was determined according to the American Joint Committee on Cancer (AJCC)/Union Internationale Contre le Cancer (UICC) classification (2009). The patients were assigned to a metastatic (32 patients) or nonmetastatic (28 patients) disease group based on pleural cavity examination and histological observation of micronodules bordering the cancer. Clinical follow-up of 38 patients was available for a mean post-surgical period of 14 months (range, 4–24); follow-up data were obtained by means of direct patient contact at 2-month intervals for the first 2 years and 4-month intervals thereafter. At the time of the last follow-up, 11 patients had died from cancer-related causes. The clinicopathological characteristics of the study patients are shown in Table I.

### Cell lines

Highly invasive lung adenocarcinoma cell line A549 was preserved in the Pathology Department of our hospital. Highly invasive 95D and poorly invasive SPC-A-1 lung adenocarcinoma cell lines were purchased from KeyGEN (KeyGEN Biotechnology Company, Nanjing, China). The three cell lines were grown in DMEM supplemented with 10% fetal bovine serum (FBS), 50 U/ml penicillin and 50 μg/ml streptomycin at 37°C with 5% CO_2_.

### Immunohistochemistry

The SP immunoperoxidase method was used to examine NEDD9 expression by immunostaining. Sections (5 μm) were deparaffinized in the oven at 60°C for 2 h, placed in xylene and rehydrated serially with alcohol and water. Endogenous peroxidase activity was quenched with 3% hydrogen peroxide in distilled water for 10 min, and following thorough washing in TBS, the slides were incubated with mouse monoclonal antibody against NEDD9 (1:100 dilution; Abcam, San Francisco, CA, USA) overnight at 4°C; followed by peroxidase-labeled polymer conjugate to anti-mouse immunoglobulins for 30 min at 37°C and developed using the DAB system. Positive and negative controls were included with each run.

### Immunohistochemical scoring

Each tissue section was separately evaluated by two pathologists (S.L. Li and L.H. Yan) who were unaware of the associated clinicopathological characteristics. Expression of NEDD9 was evaluated according to the ratio of positive cells per specimen and staining intensity as described in a previous study (10). The intensity of NEDD9 immunostaining (0, no signal; 1, weak; 2, moderate and 3, intense) and the percentage of positive tumor cells (<1%, 0; 2–25%,1; 26–50%, 2; 51–75%, and 3, >75%, 4) were assessed in at least five areas at a x400 magnification. In the case of heterogeneous immunostaining, the predominant pattern was used for scoring purposes. The scores of each case were multiplied to give a final score of 0, 1, 2, 3, 4, 6, 8, 9, or 12, and the tumors were finally classified as negative (−, score 0–1); weak (+, 2–4), moderate (++, 6–8), and strong (+++, 9–12). The immunostaining score in each section was evaluated.

### Fluorescent quantitative reverse transcription-polymerase chain reaction (FQ-PCR)

NEDD9 mRNA levels were quantified by FQ-PCR based on TaqMan™ technology, using the ABI PRISM 7500 Sequence Detection System (Applied Biosystems, Foster City, CA, USA) as described in a previous study (11). The amount of NEDD9 mRNA was normalized to an endogenous reference (GAPDH) and expressed as n-fold NEDD9 mRNA levels in relation to a calibrator. The amount of target was determined by applying the ΔΔCt method (Applied Biosystems Sequence Detector User Bulletin #2).

### RNA extraction and cDNA synthesis

Total RNA of the three cell lines was isolated using Trizol reagent (Invitrogen, Carlsbad, CA, USA) following the manufacturer’s instructions. The amount of RNA in the sample was quantified spectrophotometrically. First-strand cDNA was synthesized with the RevertAid 1st strand cDNA Synthesis kit (Fermentas, Japan) according to the manufacturer’s protocol, using 2 μg of total RNA.

### Primers and probes

The primers and TaqMan™ probes for NEDD9 and GAPDH were designed using a built-in PCR primer design software (PE Biosystems) and synthesized by a third party (Shanghai GeneCore BioTechnologies, China). The nucleotide sequences of the primers and probes are shown in Table II.

### Fluorescent quantitational PCR conditions

All FQ-PCR reactions were performed in a 25 μl mixture containing 2.5 μl Taqman fluorescent probe (2.5 μmol/l), 12.5 μl rTaqMix (2x), 0.5 μl ROX (50x), 3.5 μl Mg (25 mM), 1 μl of each upstream and downstream primer (10 μmol/l), 1 μl of cDNA template, and 3 μl ddH_2_O. FQ-PCR was performed under the following cycling conditions: 95°C force-degeneration for 2 min; 95°C degeneration for 15 sec; 60°C renaturation for 30 sec; and 60°C extension for 30 sec (40 cycles). Reactions were performed in duplicate.

### Western blotting

The cells were washed twice with cold phosphate-buffered solution (PBS) prior to being lysed in cell lysis buffer, which contained 50 mmol/l of Tris-HCl, 150 mmol/l of NaCl, 50 mmol/l of ethylene diamine tetraacetic acid (EDTA), 1% NP-40, 0.5 mmol/l of phenylmethyl sulfonylfluoride (PMSF) and 2 mg/ml of pepstatin A. The proteins, 20 μg/lane, were separated by 10% sodium dodecyl sulfatepolyacrylamide gel electrophoresis (SDS-PAGE), and then transferred to nitrocellulose membranes (Sigma, Shanghai, China). Non-specific binding sites were blocked by incubating in PBS containing 5% nonfat milk for 2 h at 37°C. Membranes were then incubated with primary antibodies overnight at 4°C. The membranes were washed 5x for 4 min with PBS-Tween 20 (PBST) and incubated with secondary antibody for 1 h at 37°C. Immunoblotting antibodies used were as follows: mouse-anti-human polyclonal antibodies NEDD9 (93 kDa, Abcam, San Francisco, CA, USA) diluted 1:500 as the primary antibody and goat-anti-mouse (IgG, Santa Cruz Biotechnology, Santa Cruz, CA, USA) diluted 1:1000 as the secondary antibody. Goat anti-β-actin polyclonal antibody (42 kDa, Santa Cruz Biotechnology) at a 1:500 dilution was employed as an internal control. Rabbit-anti-goat secondary antibody at a dilution of 1:1000 was subsequently used. Following washing of the membranes 5x for 4 min in PBST, enhanced chemiluminescence detection of the target protein was performed. Optical densities were measured using a Luminescent Image Analyzer (FujiFilm LAS-3000; Fuji Photo Film Co., Tokyo, Japan).

### Statistical analyses

All data are expressed as the mean ± standard deviation (SD). Data from two groups were analyzed by the Student’s t-test using SPSS 19.0 (SPSS Inc., USA). The survival rate was analyzed using the Kaplan-Meier method. The prognostic influence of variables on survival was analyzed using the Log-rank test for univariate analyses. A value of p<0.05 was considered statistically significant.

## Results

### Immunohistochemical analysis of NEDD9 expression in human lung adenocarcinoma tissues

**Figure 1:**
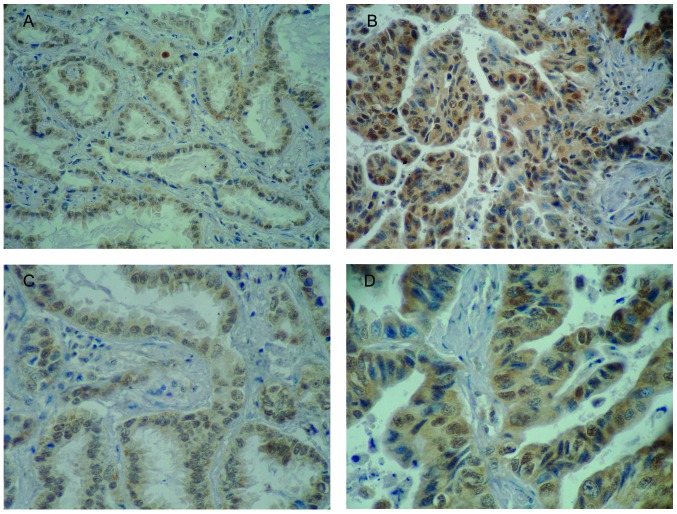
NEDD9 expression in the (A and C) nonmetastatic and (B and D) metastatic lung adenocarcinoma tissues. Original magnification: A and B, x200; C and D, x400.

NEDD9 was found to be expressed in the cell nuclei and the cytoplasm of lung adenocarcinoma cells (Fig. 1).

To better quantify the differences, five randomly chosen microscopic fields from each section were captured, and NEDD9 expression was measured as the mean stained area. The immunostaining scores revealed a statistically significant difference between the metastatic and nonmetastatic lung adenocarcinoma; the metastatic samples had an average staining score of 7±2.86 and the nonmetastatic samples had a score of 5±2.67 (p<0.001) as shown in Table III and Fig. 1.

### Quantitative evaluation of NEDD9 mRNA by FQ-PCR

The levels of NEDD9 in the highly invasive lung adenocarcinoma cell lines (A549 and 95D) were high, whereas the level of NEDD9 in the poorly invasive cell line (SPC-A-1) was low. According to FQ-PCR, highly invasive lung adenocarcinoma cell lines demonstrated 10- to 100-fold overexpression of NEDD9 mRNA relative to the less invasive cell line (Table IV).

### Western blotting of NEDD9 protein expression

**Figure 2:**
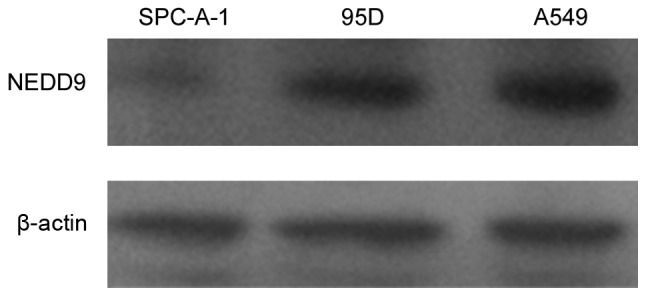
Expression of NEDD9. Western blotting of NEDD9 and β-actin in lung adenocarcinoma cell lines (SPC-A-1, 95D and A549).

Western blotting was used to evaluate NEDD9 protein expression in three lung adenocarcinoma cell lines. NEDD9 expression was significantly increased in highly invasive lung adenocarcinoma cell lines A549 and 95D. The western blotting results for the three samples are shown in Fig. 2.

### Comparative analyses and clinicopathological correlation

**Figure 3:**
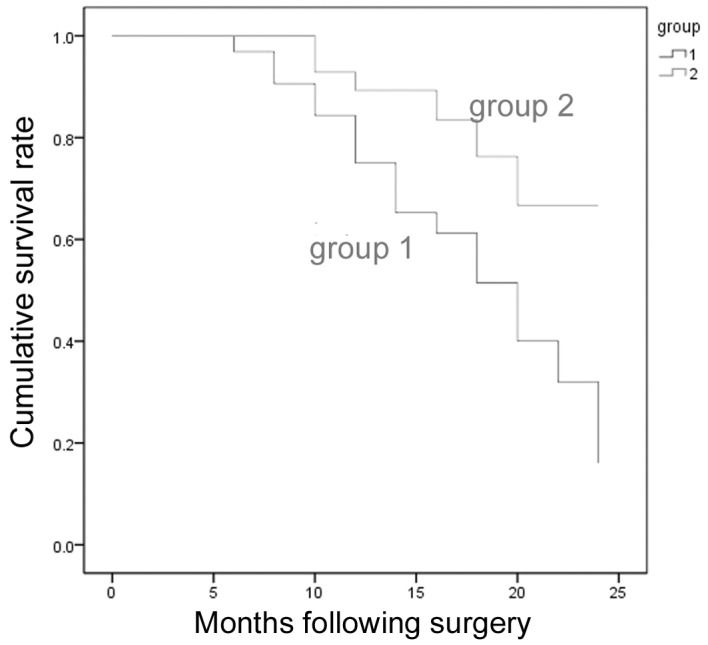
The survival curve of two patient groups. Group 1, metastatic lung adenocarcinoma cases; group 2, nonmetastatic lung adenocarcinoma cases.

The western blotting and immunohistochemical evaluations of NEDD9 expression were comparable; the lung adenocarcinoma cases with a high level of NEDD9 protein content also demonstrated intense immunostaining. Considering the NEDD9 protein expression values, immunohistochemical scoring revealed a statistically significant correlation between metastatic and nonmetastatic lung adenocarcinoma (7±2.86 versus 5±2.67, p<0.001). We compared the overall survival (OS) of the nonmetastatic lung adenocarcinoma patients and metastatic lung adenocarcinoma patients. There was a significant difference between the groups (log-rank test, p=0.026) (Fig. 3). The OS rate of the nonmetastatic lung adenocarcinoma patients was 89.3% at 1 year; 82.1.3% at 2 years. The OS rate of the metastatic lung adenocarcinoma patients was 78.1% at 1 year; 43.8% at 2 years.

## Discussion

NEDD9 is a member of the CAS (Crk-associated substrate) family. Although it lacks any known enzymatic function, it contains several functional modules for protein interaction, leading to its classification as a scaffolding protein. Overexpression of NEDD9 has variously been reported as pro-migratory (5,8,12). For example, Minn *et al* used an invasive TGF-β stimulated MDA-MB-231 cell line and a high-throughput Affymetrix assay to analyze mRNA expression and establish a gene signature associated with increased breast cancer metastasis to lung. This study indicated that a 3-fold downregulation of NEDD9 was part of the metastatic signature (6). However, analyzing genes and proteins expressed in MCF-7 breast adenocarcinoma cells upon activation of the ErbB receptor with its ligand heregulin, NEDD9 emerged as one of only five hits upregulated and hyperphosphorylated in both transcriptome and proteome datasets (13).

The NEDD9 protein is tyrosine phosphorylated by FAK and Src during cell attachment to the extracellular matrix (2,14,15). The interaction of NEDD9 and FAK is a significant onset event of cell migration and invasion (16). NEDD9 enhanced invasion *in vitro* and metastasis *in vivo* of normal and transformed melanocytes, functionally interacted with focal adhesion kinase and modulated focal contact formation and exhibited frequent robust overexpression in human metastatic melanoma relative to primary melanoma (5). Natarajan *et al* suggested that HEF1 acts as a necessary and specific downstream effector of FAK in the invasive behavior of glioblastoma cells and may be an effective target for treatment of these tumors (8). The exact mechanism of NEDD9 action in metastasis requires further investigation.

Lung cancer metastasis is a complex process with participation of multiple genes and numerous steps, among which the ‘intimate relationship’ between cancer cells and the extracellular matrix is a significant event for tumor metastasis. In human lung adenocarcinoma, NEDD9 expression is upregulated upon loss of the tumor suppressor serine/threonine kinase 11 (STK11, also called LKB1), and its expression is downregulation upon re-expression of LKB1 (17). This study describes NEDD9 expression in lung adenocarcinoma with and without metastasis for the first time. We analyzed 60 tumors by means of immunohistochemistry, three invasive lung adenocarcinoma cell lines were analyzed by FQ-PCR and western blotting, and provided immunohistochemical and molecular evidence of NEDD9 upregulation in lung adencarcinoma. Metastatic lung adenocarcinoma tissue had stronger immunostaining scores than nonmetastatic tissue. This positive correlation between NEDD9 expression and metastasis occurrence has also been widely reported in cancer and further underlines the role of this molecule in the metastatic process (5). FQ-PCR and western blotting revealed that the level of NEDD9 was markedly higher in A549 and 95D cancer cells, suggesting that NEDD9 might be involved in human lung cancer metastasis.

NEDD9 protein concentrates at focal adhesions, the centrosome and mitotic spindle, although cytoplasmic pools of proteins exist (1,18,19). We analyzed 60 lung adenocarcinoma tissues with or without metastases by means of immunohistochemistry. NEDD9 was found to be expressed in the cell nuclei and the cytoplasm of the lung adenocarcinoma cells. The possible reason that NEDD9 is strongly regulated by the cell cycle may be that NEDD9 protein levels are very low in quiescent or G1 populations, increase during the S phase, and reach peak abundance in late G2/M (20). A cell cycle specificity agent triazin (named A190) that inhibits the NEDD9 signal transduction pathway has proven to be useful *in vivo* on nude mice xenografts (21). Taken together, these findings suggest that NEDD9 upregulation may be an early event in lung neoplastic transformation and further support previously published data indicating its ability to mediate cell spreading and to play a significant role in driving cell migration (22).

NEDD9 has a molecular weight of 93 kDa, and migrates as a doublet of 105 and 115 kDa (23). Its apparent high molecular weight is largely due to its extensive phosphorylation. Drugs that target FAK, Src, BCR-ABL, and TGF-β are already in clinical use, and may be particularly effective in tumors overexpressing NEDD9 through limiting NEDD9 phosphorylation.

In conclusion, we proved that NEDD9 was markedly upregulated in human metastatic lung adenocarcinoma tissues and highly invasive lung adenocarcinoma cell lines. Increasing the expression of NEDD9 may lead to an increase in lung cancer cell invasion ability. The findings suggest that NEDD9 may be closely related to metastasis of lung adenocarcinoma. Future studies should use siRNA to inhibit the expression of NEDD9 *in vitro* and *in vivo*.

## Figures and Tables

**Table I:** Clinicopathological characteristics of the patients and tumors.

Characteristics	Tissues	
	without metastases n (%)	with metastases n (%)
No. of patients	28	32
Mean age (years)	58±10.5	60±8.9
Gender		
Male	20 (71)	22 (69)
Female	8 (29)	10 (31)
Tumor differentiation		
High	3 (11)	0 (0)
Middle	18 (64)	18 (56)
Low	7 (25)	14 (44)
Tumor grade		
T1	4 (15)	3 (9)
T2	10 (35)	11 (34)
T3	10 (35)	11 (34)
T4	4 (15)	7 (23)
Stage grouping		
I	11 (39)	1 (3)
II	12 (43)	10 (31)
III	4 (14)	15 (47)
IV	1 (4)	6 (19)

**Table II:** The primer and probe sequences of the NEDD9 and GAPDH genes.

Gene name		Sequence
NEDD9 (124 bp)	Forward	5′-CGTGGGTAAAAAGGTGTTCC-3′
	Reverse	5′-CAAGCCTCCAAACTCAGGAC-3′
	Probe	5′-(6-Fam)-CAAACCAGCTTGTGAACCTCCAC-(Tamra)-3′
GAPDH (97 bp)	Forward	5′-TCGTGGAAGGACTCATGACC-3′
	Reverse	5′-AGGGATGATGTTCTGGAGAG-3′
	Probe	5′-(6-Fam)-CCATCACTGCCACCCAGAAGAC-(Tamra)-3′

**Table III:** NEDD9 protein expression in lung adenocarcinoma.

		NEDD9 immunostaining			
	Lung adenocarcinoma n (%)	−	+	++	+++
Nonmetastatic	28 (37%)	2 (7)	11 (39)	12 (43)	3 (11)
Metastatic	32 (53%)	1 (3)	8 (25)	15 (47)	8 (25)

−, negative;

+, weak;

++, moderate;

+++, strong.

See Materials and methods for tumor classification of NEDD9 immunoreactivity.

**Table IV:** Relative expression of NEDD9.

Group	ΔCt_mean_	−ΔΔCt	Fold (mean ± SD)	P-value
SPC-A-1	8.10	0	1	
A549	1.79	6.37	84.07±18.99	0.003
95-D	3.39	4.77	42.92±48.75	0.003

Fold = 2^−ΔΔCt^; ΔΔCt = (Ct_NEDD9_ - Ct_GAPDH_)_A549/95-D_ - (Ct_NEDD9_ - Ct_GAPDH_)_SPC-A-1_
